# Harnessing a Safe Novel Lipid Nanoparticle: Targeted Oral Delivery to Colonic Epithelial and Macrophage Cells in a Colitis Mouse Model

**DOI:** 10.3390/nano14221800

**Published:** 2024-11-09

**Authors:** Rabeya Jafrin Mow, Michal Pawel Kuczma, Xiaodi Shi, Sridhar Mani, Didier Merlin, Chunhua Yang

**Affiliations:** 1 Digestive Disease Research Group, Institute for Biomedical Sciences, Georgia State University, Atlanta, GA 30303, USA; rmow1@student.gsu.edu (R.J.M.); mkuczma@gsu.edu (M.P.K.); xshi10@student.gsu.edu (X.S.); or didier.merlin@va.gov (D.M.); 2 Albert Einstein College of Medicine, Bronx, NY 10461, USA; sridhar.mani@einsteinmed.edu; 3 Atlanta Veterans Affairs Medical Center, Decatur, GA 30302, USA

**Keywords:** Inflammatory Bowel Disease (IBD), macrophage-targeting, lipid labeling, colonic epithelium, flow cytometry

## Abstract

A novel lipid nanoparticle (nLNP), formulated with three essential lipids to mimic ginger-derived exosomal particles, shows strong potential for delivering IL-22 mRNA specifically to the colon, presenting a unique oral drug delivery system for inflammatory bowel disease (IBD). However, its cellular targets and uptake behavior in healthy versus diseased colons remain unclear. Understanding these aspects is crucial for fully elucidating its targeting effectiveness in inflamed colon tissue. This study investigates the nLNP’s cellular targets in healthy and diseased mouse colons. Flow cytometry compared nLNP uptake in healthy mice and a DSS-induced acute colitis model. The results revealed efficient internalization of nLNP by colonic epithelial cells in healthy and inflamed mice. In non-inflamed mice, the small number of colonic macrophages resulted in minimal uptake of nLNP by these cells. In inflamed mice, macrophages migrated to the damaged epithelium, where nLNP uptake was significantly increased, highlighting the nLNP’s ability to target both epithelial and macrophage cells during inflammation. Additionally, safety assessments showed that the nLNP neither altered in vitro kinase activities nor exhibited immunotoxicity or induced in vivo toxicity at the maximum tolerated oral dose. These findings underscore the nLNP’s safety and potential as a promising epithelial/macrophage-targeted drug delivery platform for oral ulcerative colitis (UC) treatment.

## 1. Introduction

Inflammatory bowel disease (IBD) is a group of chronic conditions characterized by recurring inflammation and damage to the gastrointestinal (GI) tract [1]. The two primary forms of IBD, Crohn’s disease (CD) and ulcerative colitis (UC), are both marked by persistent intestinal inflammation and epithelial injury [2]. The prevalence of IBD has been rising steadily, particularly in developed countries [3,4]. Although its precise etiology is not fully understood, IBD is believed to involve a complex interplay of genetic, environmental, and immunological factors [5]. Recent advancements in understanding IBD pathophysiology have led to the development of novel diagnostic and therapeutic strategies, improving disease management and patient outcomes [6,7,8].

Conventional treatments for UC, including anti-inflammatory drugs, immunosuppressants, and biologics, aim to reduce inflammation and induce remission. However, these therapies often require systemic administration at high doses, leading to severe side effects. For instance, anti-inflammatory drugs such as 5-aminosalicylic acid (5-ASA) derivatives are commonly used but can cause nephrotoxicity and gastrointestinal complications, especially at higher doses [9,10]. Immunosuppressants such as azathioprine and mercaptopurine are mainstream therapeutics for remission but pose significant risks, including leukopenia, increased susceptibility to infections, and hepatotoxicity [11,12]. Biologics, especially anti-TNF antibodies like infliximab and adalimumab, carry the risk of severe diseases, such as tuberculosis, and an increased likelihood of malignancies, including lymphoma [13,14,15]. These challenges highlight the urgent need for targeted delivery systems capable of administering precise, low doses of medication directly to inflamed colonic regions over an extended period.

In response to the need for more precise, targeted UC treatments, lipid nanoparticles (LNPs)—particularly those derived from plants—have emerged as a promising drug delivery modality. These plant-derived LNPs enhance drug stability, improve targeting of diseased tissues, and control drug release [16,17]. Their small size, unique membrane composition, and ability to encapsulate both hydrophilic and hydrophobic drugs enable them to protect therapeutic agents from enzymatic degradation in the gastrointestinal tract and enhance absorption across biological membranes [18,19]. This makes plant-derived LNPs particularly effective for targeted therapeutic delivery to the colon, representing a significant advancement over conventional treatments that often impact the entire body [20]. Other bio-inspired nanovesicles are also gaining traction as researchers explore nature for innovative solutions that offer biocompatibility, targeted delivery, and immune evasion to address drug delivery challenges [21,22]. For example, glioblastoma-cell membrane-derived nanovesicles have shown potential for tumor-specific drug delivery, providing a model for precision therapies in complex diseases [23]. Additionally, bioinspired vesicles offer expanded functionalization options, enabling researchers to leverage diverse biomimetic sources to optimize both therapeutic and diagnostic payloads [24].

Drawing inspiration from the colon-targeting ability of ginger-derived exosomal nanoparticles, our previous research successfully developed a novel lipid nanoparticle (nLNP) capable of encapsulating and delivering IL-22 mRNA specifically to the colon. This nLNP is composed of three key lipids—DGDG, MGDG, and PA—in the 3:2:5 ratio found in GDNPs, collectively representing over 90% of the total lipid content in GDNPs [1]. By using these pure lipids, this nature-inspired nLNP eliminates the other components of GDNPs (such as proteins, miRNAs, gingerols, and shogaols) while preserving the physical characteristics and colon-targeting properties of GDNPs, demonstrating a well-defined nLNP with a significant ability to deliver therapeutics to alleviate UC symptoms in earlier studies [25].

Building on these promising results, this study explores the cellular targets of nLNP in inflamed and non-inflamed conditions to deepen our understanding of their targeting efficiency and therapeutic efficacy. By examining how nanoparticle uptake differs between healthy and diseased states, we aim to predict its performance in pathological conditions. Additionally, we conducted a comprehensive safety evaluation of the nLNP, assessing potential cytotoxicity, immunotoxicity, and overall tolerability in both in vitro and in vivo settings. These insights will help mitigate clinical risks and support the future development of nLNP for clinical trials.

## 2. Materials and Methods

### 2.1. Materials and Instruments

Digalactosyldiacylglycerol (DGDG), Monogalactosyldiacylglycerol (MGDG), and L-α-phosphatidic acid (PA) were purchased from Avanti Polar Lipids (Birmingham, AL, USA) and stored at −20 °C. Ethanol (200 proof) was obtained from Decon Labs (King of Prussia, PA, USA). Dichloromethane (DCM) and Amicon^®^ Ultra 15 mL centrifugal filter (Ultracel^®^-100k) were purchased from Sigma Aldrich (St. Louis, MO, USA). 4-Di-16-ASP 4-(4-(Dihexadecylamino)styryl)-N-Methylpyridinium Iodide (DiA) and Dimethyl sulfoxide (DMSO) were sourced from Thermo Fisher Scientific (Rockford, IL, USA). Phosphate-buffered saline (PBS) was purchased from Corning (Manassas, VA, USA). Roswell Park Memorial Institute Medium (RPMI), Advanced Dulbecco’s Modified Eagle Medium (DMEM/F-12), HEPES, GlutaMAX, Penicillin-Streptomycin (Pen/Strep, 10,000 U/mL) and EDTA (0.5 M, pH 8.0) were purchased from Thermo Fisher Scientific (Rockford, IL, USA). HBSS was obtained from Cytiva (Marlborough, MA, USA). DNase I was sourced from Roche (Indianapolis, IN, USA). Liberase^TM^ was obtained from Sigma Aldrich (St. Louis, MO, USA). TrypLE was purchased from Invitrogen (Waltham, MA, USA). Dextran sodium sulfate (DSS) was acquired from MP Biomedicals, LLC (Santa Ana, CA, USA). Trypan Blue solution (0.4%) was purchased from Thermo Fisher Scientific (Rockford, IL, USA). 4′,6-diamidino-2-phenylindole (DAPI), CD45 (Alexa Fluor 700), EpCAM (APC-eFluor 780), and F4/80 (APC) were purchased from Thermo Fisher Scientific (Rockford, IL, USA). Ly6C (BV 605) and CD11b (BV 780) were sourced from BioLegend (San Diego, CA, USA). 2.4G2 was purchased from BioXcell (Leba-non, NH, USA). FBS was obtained from R&D Systems (Minneapolis, MN, USA). Sodium Azide was purchased from Teknova (Hollister, CA, USA).

Nanoparticle size and surface potential were obtained on Zetasizer (Malvern Panalytical, Westborough, MA, USA), and nanoparticle morphology was visualized by CoreAFM atomic force microscopy (Nanosurf, Liestal, Switzerland). Cell population and live cell percentage were measured using Countess 3 FL Automated Cell Counter (Invitrogen, Carlsbad, CA, USA). A flow cytometry study was performed on Cytoflex LX (Beckman, San Jose, CA, USA).

### 2.2. Preparation and Characterization of nLNP

To prepare the nLNP, we made lipid solutions by dissolving 10 mg Digalactosyldiacylglycerol (DGDG) in 5 mL 200 proof ethanol (2 mg/mL) and 10 mg Monogalactosyldiacylglycerol (MGDG) in 5 mL 200 proof ethanol (2 mg/mL) and dissolving 25 mg phosphatidic acid (PA) in 5 mL dichloromethane (DCM) to generate a 5 mg/mL PA solution. Then, these solutions were combined in a pear-shaped flask and homogenized by adding an extra 200-proof ethanol. The lipid mixture was then subjected to rotary evaporation to remove all the organic solvents and to form a thin lipid film. Phosphate-buffered saline (PBS) was subsequently added to initiate hydrolysis. The mixture was then sonicated in an ultrasonic bath (55–60 °C) with intermittent pipetting for approximately 5 min until a homogenized translucent suspension was observed, indicating the formation of nLNP [25]. The resulting nLNP suspension was stored at 4 °C for further characterization.

The particle size and surface zeta potential of the new lipid nanoparticles (nLNP) were measured by dynamic light scattering using Zetasizer (Malvern Panalytical, Westborough, MA, USA). The final lipid concentration used for DLS analysis was 0.2 mg/mL. Measurements were performed at 25 °C, with each sample measured in triplicate to ensure reproducibility. Morphological images of the nLNP were obtained using CoreAFM atomic force microscopy (Nanosurf, Liestal, Switzerland). For AFM sample preparation, the nLNP solution was diluted in ddH_2_O to a concentration of 0.02 mg/mL, and 2 μL was deposited onto a mica sheet. The sample was air-dried at room temperature for 2 h before imaging. The detailed protocol is available in the published bio-protocol paper [26].

### 2.3. Biodistribution and Colon-Targeting Efficiency

We labeled the nLNP with lipophilic fluorescent dye DiA to investigate the biodistribution and colon-targeting efficiency. Briefly, a 10 µL aliquot of DiA/DMSO solution (5 mg/mL) was added to 5 mL of nLNP PBS suspension, resulting in a final concentration of 10 µg/mL for the fluorescent dye DiA in the nLNP solution. The mixture was then covered with aluminum foil and incubated at room temperature for 15 min with continuous shaking at 100 rpm. Following incubation, the nLNP suspension was transferred to centrifugal filters (molecular weight cut-off: 100 kDa) and centrifuged at ~4500× *g* for 10 min at 10 °C to remove the free dye. The retained nLNP was reconstituted in PBS. Mice were orally administered 0.2 mL of the DiA-labeled nLNP suspension. After 24 h, the mice were sacrificed, and their gastrointestinal tracts were harvested for imaging using the in vivo imaging system (IVIS). Detailed procedures can be found in the previously published bio-protocol [27]. 

### 2.4. DSS-Induced Acute Colitis Mouse Model

Female C57BL/6 mice (six to seven weeks old) were purchased from Jackson Laboratory (Bar Harbor, ME, USA). The mice were acclimatized in a specific pathogen-free environment under controlled conditions (22 ± 2 °C). All animal experiments were carried out by guidelines evaluated and approved by the institutional animal care and use committee (IACUC, protocol #23033) of Georgia State University (Atlanta, GA, USA). Acute colitis was induced in female C57BL/6 mice by administering 2% dextran sodium sulfate (DSS) in their drinking water for seven days. The DSS solution was freshly prepared every two days. After the seven-day treatment period, the mice were orally administered DiA-labeled nLNP, then acclimated in the cage for 24 h. and euthanized using 4% CO_2_, followed by cervical dislocation. 

### 2.5. Identification of Cellular Targets

#### 2.5.1. Isolation of Cells from Mouse Colon Tissues

Colon tissues were harvested from healthy and DSS-treated mice. The colons were excised, cleaned, cut longitudinally, and washed three to five times with ice-cold HBSS until fecal materials were thoroughly removed. The tissue was then cut into approximately 2 cm long segments and kept on ice until further processing.

To isolate crypts, we transferred colon segments to a 50 mL conical tube containing 15 mL of epithelial cell solution [28]. We incubated them at 37 °C in a water bath for 15 min, with gentle shaking every 5 min. After incubation, the tubes were placed on ice for 10 min. The tissue was transferred to a new 50 mL conical tube with fresh HBSS and shaken vigorously for 30 to 40 s. The supernatant containing crypts was filtered through a 100 μm strainer and centrifuged, and the crypt pellet was resuspended in epithelial wash buffer [28] and kept on ice. The remaining tissue, representing the lamina propria, was transferred to a 50 mL conical tube with 20 mL of HBSS for further digestion [28].

For lamina propria isolation, the remaining tissue was washed three times with HBSS and cut into smaller pieces (~0.5 cm). The pieces were transferred to a 15 mL conical tube with lamina propria solution [28] and incubated at 37 °C in a water bath for 20 min. The tissue was then mechanically dissociated by pipetting until it passed smoothly through the pipette. After an additional 10-min incubation at 37 °C, further mechanical dissociation was performed until complete tissue digestion. The digestion was stopped by adding 1 mL of FBS and 80 μL of EDTA. The resulting cell suspension was filtered through a 40 μm strainer, washed with HBSS, and centrifuged (~250× *g*). The cell pellet was resuspended in RPMI. 

The epithelial fraction (containing crypts) was spun down and resuspended in 5 mL of pre-warmed TrypLE with DNase I (100 μg/mL), followed by gentle pipetting. The reaction was stopped with 1 mL of FBS, and the solution was filtered through a 40 μm strainer, with epithelial wash buffer added to 50 mL. After centrifugation, the pellet was resuspended in an epithelial wash buffer [28]. 

Cell viability and count were assessed using trypan blue (0.4%) staining and a cell counter to ensure the isolated cells were viable and suitable for downstream applications.

#### 2.5.2. Flow Cytometry Analysis

Cell preparation was done as reported [29]. Briefly, spleens were mechanically disrupted and filtered through 40 μm mesh. Splenic RBCs were lysed with ACK buffer. For flow cytometry analysis, 1 × 10^6^ cells were used for staining. FcR was blocked with 2.4G2 antibody (10 min, RT; BioXcell), and cells were subsequently labeled with titrated fluorochrome-tagged mAbs (20 min, RT; Thermo, BioLegend) in FACS buffer (PBS/1% (*v*/*v*) FBS/0.1% (*w*/*v*) sodium azide). Cells were washed with PBS (500× *g*, 1 min) and resuspended in FACS buffer for analysis. Dead cells were excluded with DAPI. Cells were acquired on Cytoflex LX (N3-V5-B3-Y5-R3-I2; 6 lasers (375 nm, 405 nm, 488 nm, 561 nm, 638 nm, 808 nm), 21 parameters; Beckman) equipped with plate loader for an automatic sample acquisition. At least 1 × 10^5^ live cells were acquired. Data was analyzed with FlowJo (v.10.10.0; BD, Ashland, OR, USA) and Prism (v.10.1.2; GraphPad, San Diego, CA, USA). For tSNE analysis, the following plugins were used to generate FlowJo plots with 5 × 10^4^ live cells per sample: DownSampleV3, FlowAI, FlowSOM, FlowClean, FlowMeans.

### 2.6. Safety Evaluation of nLNP

#### 2.6.1. In Vitro Evaluation of Safety and Toxicity (InVEST) Panel 

An in vitro safety and toxicity assessment of nLNP was conducted to evaluate its safety profile for potential use as an oral drug carrier. The evaluation included a panel of assays to test the impact of nLNP on 19 biological targets, including G-protein-coupled receptors (GPCRs) (A1 adenosine, A2A adenosine, α1A adrenergic, α2A adrenergic, β1 adrenergic, CCK, D1 dopamine, D3 dopamine, Muscarinic M1, Muscarinic M2, Muscarinic M3, 5-HT1A, 5-HT1B, 5-HT2A, 5-HT3), a nuclear receptor (AHR), phosphodiesterases (PDE3A and PDE4A), and a protease (Thrombin α). These targets are associated with human toxicity, as their inhibition may result in serious health risks. Catalytic assays were used for phosphodiesterases, radioligand binding assays were used for GPCRs, and cell-based transcriptional assays were employed for AHR activity. nLNP was tested at 20 μM in duplicate, while reference compounds were evaluated using 8- or 10-point concentration-response curves with varying starting concentrations.

#### 2.6.2. Immunosafety Evaluation in Human Immune Cells

We thawed frozen human peripheral blood mononuclear cells (PBMCs) and suspended them in T-cell expansion media at a concentration of 1 × 10^7^ cells per mL. After transferring the cells to a T75 flask, we incubated them overnight at 37 °C with 5% CO_2_. The following day, we labeled the cells with a proliferation dye, re-suspended them in fresh T-cell expansion media, and plated them in 96-well plates at a density of 1 × 10^4^ cells per well, followed by overnight incubation. To prepare the test compounds, we dissolved lyophilized nLNP powders in DMSO and diluted them 1000-fold with culture media to achieve 5, 20, and 100 µM final concentrations. We incubated the cells with the nLNP solution at 37 °C with 5% CO_2_ for four days. For controls, we treated cells with either blank culture media (standard control) or culture media containing 0.1% DMSO (solvent control, SC). Cells stimulated with a CD3/CD28 T cell activator (25 μL/mL) served as the positive control. Interleukin-2 (IL-2) was added to selected groups to stimulate the immune response, while other groups were left untreated. Triplicate samples were prepared for each condition. After four days of incubation, cells were harvested and stained with antibodies targeting CD4 [allophycocyanin (APC)] and CD8 (APC/Cy7), along with the viability dye 7-Amino-Actinomycin D (7-AAD). Flow cytometry was used to analyze the samples, quantifying the percentage of viable CD4+ and CD8+ T cell subsets and assessing the proliferative response of both CD4+ and CD8+ cells.

#### 2.6.3. Maximum Tolerated Dose (MTD)

We also conducted a general toxicity study to evaluate the safety of nLNP using the maximum tolerated dose (MTD) in 10-week-old CD-1 mice. Mice received an oral administration of 200 mg/kg of nLNP for five consecutive days, reaching a cumulative dose of 1000 mg/kg, aligning with FDA guidelines for MTD studies. A control group received PBS. Over a seven-day post-dose observation period, we monitored the mice for signs of toxicity, including changes in body weight and clinical scores based on activity, physical appearance, and overall body condition. This assessment allowed us to evaluate the potential systemic toxicity of nLNP at a high dose. In addition to physical and behavioral assessments, we performed hematological and biochemical analyses to assess systemic toxicity further. Blood samples were collected to measure white blood cell count, red blood cell count, hemoglobin levels, and critical parameters related to hepatic and renal function.

### 2.7. Statistical Analysis

Data visualization and analysis were done using GraphPad Prism 9 and Microsoft Excel 2013. The results represent biological replicates, and outlier detection was performed using GraphPad’s outlier calculator with an alpha threshold of 0.05. Statistical significance was assessed through unpaired two-tailed Student’s *t*-test or one-way ANOVA, with significance levels indicated as follows: * *p* < 0.05 and ** *p* < 0.01.

## 3. Results

### 3.1. Formulation and Characterization of nLNP

**Figure 1:**
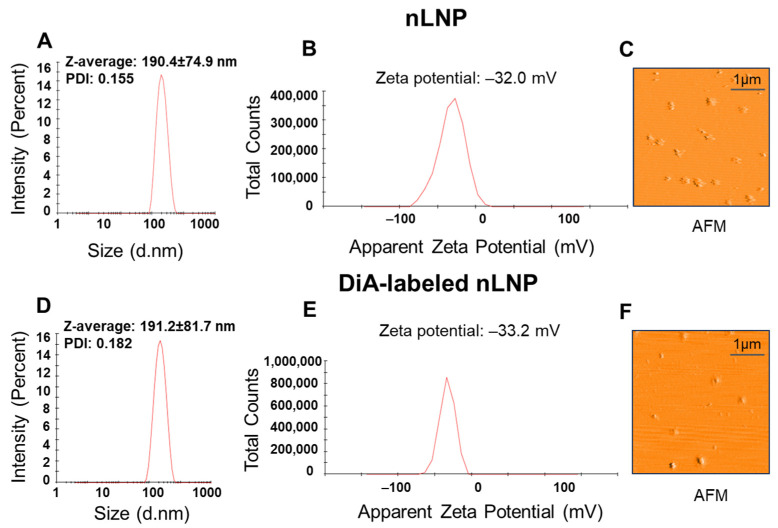
Characterization of nLNP. (**A**–**C**) Representative images showing size, zeta potential, and atomic force microscopy (AFM) results for pristine nLNP. (**D**–**F**) Representative images showing size, zeta potential, and AFM results for DiA-labeled nLNP.

Leveraging the colon-targeting properties of ginger-derived exosomal nanoparticles (GDNPs), we formulated nLNP using three essential lipids—DGDG, MGDG, and PA—combined in a 3:2:5 ratio as found in GDNPs [25]. GDNPs possess essential physical characteristics, including nanoscale size, stable negative zeta potential, and spherical morphology, which are critical for stability and targeted delivery to inflamed colonic tissues [30]. By assembling nLNP with these same properties, we aim to enhance its suitability for colon-targeted oral drug delivery. Using dynamic light scattering, we measured the particle size and surface zeta potential of both pristine and DiA-labeled nLNP. The average diameters were 190.4 nm for pristine nLNP and 191.2 nm for DiA-labeled nLNP, with corresponding polydispersity indices (PDIs) of 0.155 and 0.182 (Figure 1A,D), indicating a uniform particle size distribution. The surface zeta potentials were −32.0 mV for pristine nLNP and −33.2 mV for DiA-labeled nLNP (Figure 1B,E). Atomic force microscopy (AFM) confirmed the spherical morphology of the nLNP (Figure 1C,F). These findings suggest that DGDG, MGDG, and PA can be reassembled into stable nLNP (with or without DiA labeling) while maintaining the physical properties of GDNPs.

### 3.2. Colon-Targeting Efficiency of Orally Delivered nLNP

**Figure 2:**
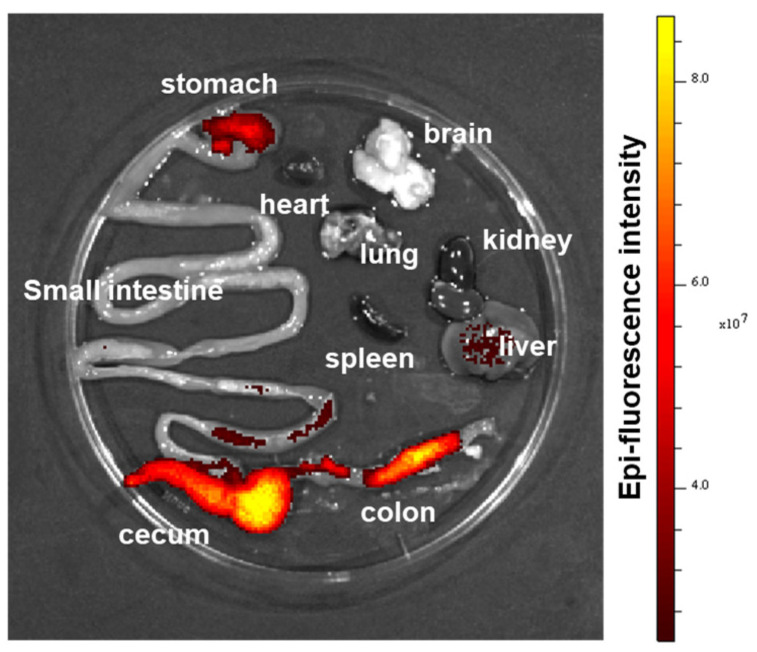
Biodistribution of DiR-tagged nLNP in mouse organs after oral administration. The representative picture shows the fluorescence detected in different mouse organs (stomach, heart, brain, small intestine, lung, kidney, spleen, liver, caecum, and colon).

To demonstrate the effectiveness of nLNP as an oral delivery platform for targeted drug delivery to the colon, we fasted the mice for 4 h before giving them 200 μL of DiA-labeled nLNP. After 2 h, food was reintroduced, and 18 h later, the mice were sacrificed, and their gastrointestinal tracts were collected for analysis using the in vivo imaging system (IVIS). Fluorescent images revealed that the orally administered nLNP was predominantly localized in the caecum and colon (Figure 2), confirming its colon-specific targeting ability, as previously demonstrated [27].

### 3.3. Cellular Targets and Uptake Behavior of nLNP

#### 3.3.1. Inflammation Reduces Epithelial Cells and Increases Immune Cells in the Colon Mucosa

**Figure 3:**
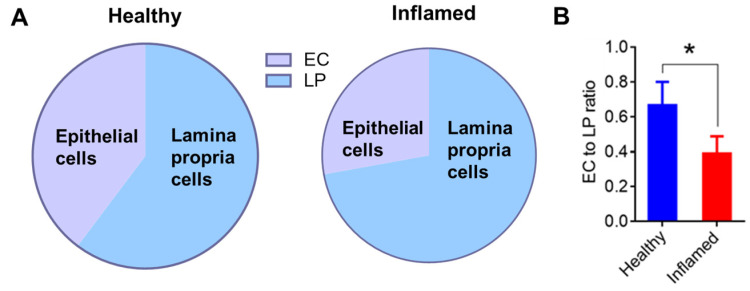
Acute colitis decreases epithelial cell numbers while increasing lamina propria cells. (**A**) Pie charts showing the distribution of cells isolated from the epithelium compared to the lamina propria layer of colon tissues. (**B**) Bar graph comparing the ratio of epithelial cells (EC) to lamina propria cells (LP) with and without inflammation. Error bars represent one standard deviation (n = 3; * *p* < 0.05).

We compared cell numbers from different colonic tissue layers dissected from healthy and inflamed mice. The results showed that DSS-induced acute colitis significantly reduced the number of epithelial cells in the epithelium layer while increasing the number of immune cells isolated from the lamina propria in inflamed mice compared to healthy mice (Figure 3). This shift in the cellular composition of the colonic mucosa indicates the impact of epithelial barrier disruption, facilitating more significant immune cell infiltration. The compromised barrier allows luminal antigens to access the lamina propria, amplifying the inflammatory response [31].

#### 3.3.2. Macrophage Migration to the Epithelium in Response to Inflammation

**Figure 4:**
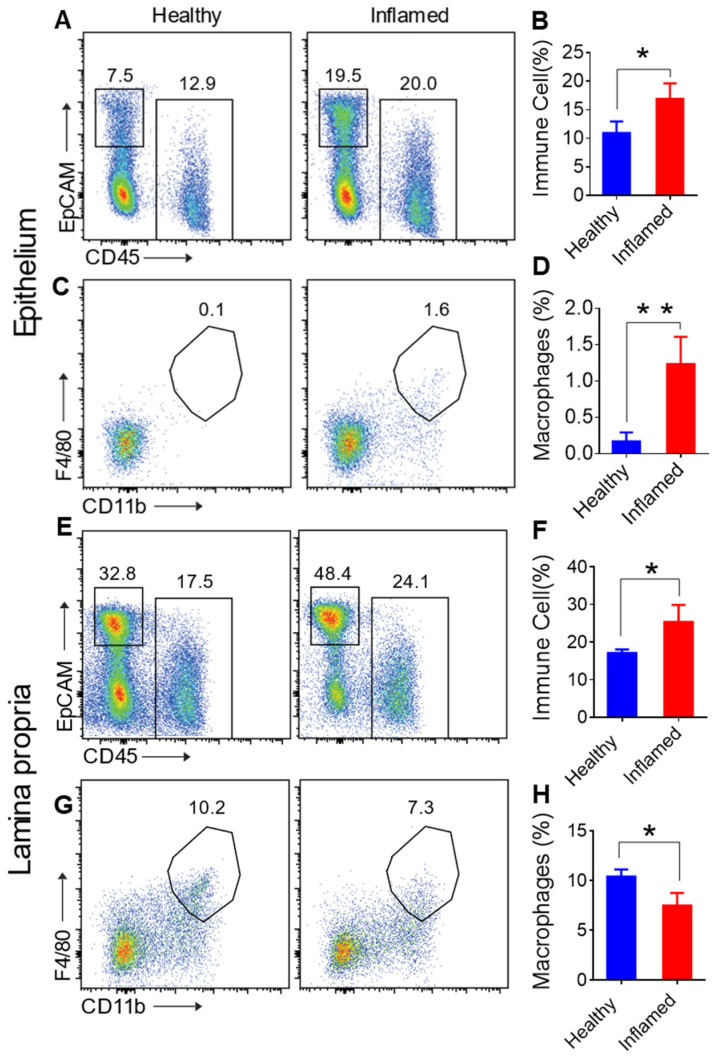
Proportions of immune cells (CD45+), epithelial cells (CD45-EpCAM+), and macrophages (CD45+CD11b+F4/80+) in cells isolated from the epithelium and lamina propria. (**A**) FACS analysis of immune cells from healthy (left) and inflamed (right) epithelium. (**B**) Comparison of immune cell proportions in the epithelium between healthy and diseased colon. (**C**) FACS analysis of macrophages from healthy (left) and inflamed (right) epithelium. (**D**) Comparison of macrophage proportions in the epithelium between healthy and diseased colon. (**E**) FACS analysis of immune cells from healthy (left) and inflamed (right) lamina propria. (**F**) Comparison of immune cell proportions in the lamina propria between healthy and diseased colon. (**G**) FACS analysis of macrophages from healthy (left) and inflamed (right) lamina propria. (**H**) Comparison of macrophage proportions in the lamina propria between healthy and diseased colon. Error bars represent one standard deviation (n = 3; * *p* < 0.05; ** *p* < 0.01).

Flow cytometry analysis further revealed a significant increase in immune cells in inflamed mice (Figure 4A,B), including macrophages in the epithelial fraction (Figure 4C,D). In contrast, while overall immune cell numbers increased in the lamina propria (Figure 4E,F), macrophage numbers decreased (Figure 4G,H). This suggests that macrophages migrate to the damaged epithelium in response to inflammation, driven by signals from inflammatory mediators and damaged cells that attract immune cells to the injury site [31]. Thus, in inflamed mice, there is an increase in immune cells, including macrophages, in the epithelial fraction. In contrast, macrophages decrease in the lamina propria despite the overall rise in immune cell numbers.

#### 3.3.3. Enhanced nLNP Uptake by Epithelial Cells and Macrophages in Inflamed Epithelium

**Figure 5:**
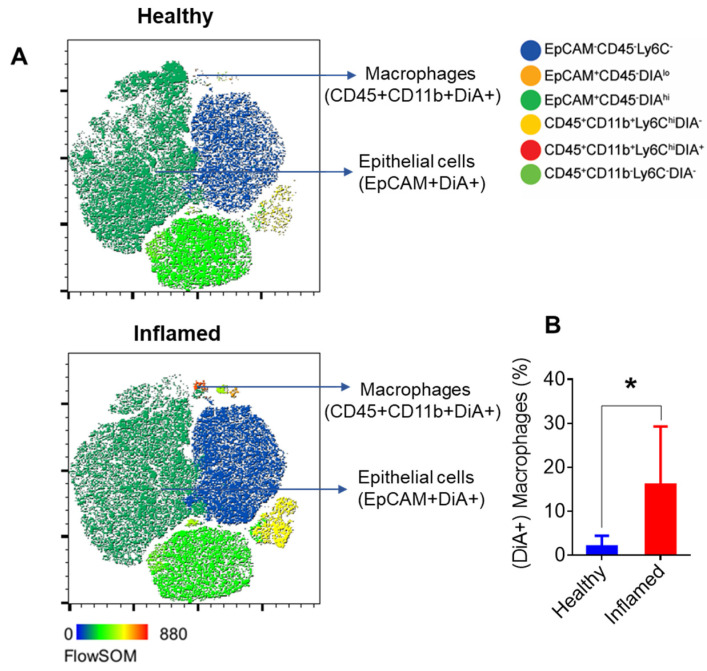
Increased nLNP uptake by macrophages in the epithelial fraction of inflamed mice. (**A**) t-SNE analysis of FACS data shows the proportion of epithelial cells (EpCAM+DiA+) and macrophages (CD45+CD11b+DiA+) that uptake DiA-labeled nLNP in healthy (**upper**) and inflamed (**lower**) tissue. (**B**) Comparison of macrophages internalizing nLNP between healthy and diseased colon epithelium. Error bars represent one standard deviation (n = 3; * *p* < 0.05).

Next, we aimed to determine which colonic cells internalized the nLNP and whether there are differences in internalization behaviors between healthy and inflamed mice. Flow cytometry analysis revealed efficient internalization of nLNP by colonic epithelial cells in healthy and inflamed mice (Figure 5A, upper t-SNE plot). In inflamed mice, macrophages migrated to the damaged epithelium, where nLNP uptake was significantly increased due to the increased number of macrophages and their enhanced uptake efficiency (Figure 5A, lower t-SNE plot, and Figure 5B). This observation suggests that nLNP offers a targeted method for delivering therapeutic agents to inflamed colonic tissue.

### 3.4. nLNP Is a Safe Oral Drug Delivery System

#### 3.4.1. Comprehensive In Vitro Toxicity Assessment of nLNP for Oral Drug Delivery

Building on the findings of cell-specific nLNP uptake in both inflamed and healthy conditions, we next evaluated the safety profile of nLNP through a comprehensive toxicity assessment. The InVEST panel is an extensive in vitro assay system designed to assess the toxicity of drug candidates, including drug delivery systems (DDS). It targets key biological pathways and cellular interactions by evaluating cytotoxicity, genotoxicity, immunotoxicity, and organ-specific effects. In this study, we employed the InVEST panel to examine the impact of nLNP on 19 toxicity-related targets, including G-protein-coupled receptors (GPCRs), nuclear receptors, phosphodiesterases (PDE3A, PDE4A), and Thrombin α. The results indicated no significant inhibition of GPCRs (90–115%), phosphodiesterases (80–101%), or Thrombin α (104%). Additionally, AHR activity remained stable, with neither activation (95%) nor inhibition (101%). Detailed enzymatic activity data are provided in Table 1.

#### 3.4.2. nLNP Is Safe for In Vitro Cultured Human Immune Cells

**Figure 6:**
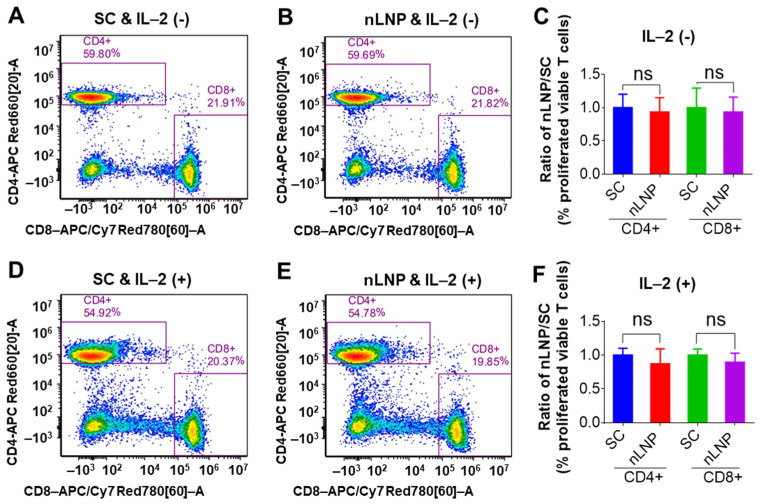
FACS analysis of cultured PBMCs, gating on viable CD4+ and CD8+ T cells in solvent control (SC) and nLNP-treated groups, with (+) or without (−) IL-2 addition. (**A**) A representative FACS image shows the percentage of viable proliferating CD4+ and CD8+ T cells in the SC group without IL-2 addition. (**B**) Percentage of viable proliferating CD4+ and CD8+ T cells in the nLNP-treated group without IL-2 addition. (**C**) Comparison of proliferated viable T cells between the nLNP-treated and SC groups without IL-2 addition. (**D**) Percentage of viable proliferating CD4+ and CD8+ T cells in SC group with IL-2 addition. (**E**) Percentage of viable proliferating CD4+ and CD8+ T cells in the nLNP-treated group with IL-2 addition. (**F**) Comparison of proliferated viable T cells between the nLNP-treated and SC group with IL-2 addition. Data are presented as mean ± SD (n = 3, ns: non-significant).

While the InVEST panel provided a broad overview of nLNP’s toxicity across various pathways, we conducted a more focused investigation into its immunosafety using peripheral blood mononuclear cells (PBMCs) isolated from three healthy human donors, as immune toxicity is a critical factor that often hampers the success of IBD drug development. Flow cytometry analysis showed that nLNP, at concentrations up to 100 µM, did not significantly affect the total number of viable human lymphocytes, including CD4+ (T helper) and CD8+ (cytotoxic T) cells, nor did it impair the proliferation of these lymphocyte subsets compared to the solvent control (SC) (Figure 6). Furthermore, its immunosafety profile was consistent in both resting PBMCs (without IL-2 activation, Figure 6A–C) and activated PBMCs (with IL-2 stimulation, Figure 6D–F). These results suggest that nLNP alone does not exhibit immunomodulatory or immunotoxic effects, indicating that this new nLNP could be a safe carrier for efficient in vivo drug delivery.

#### 3.4.3. Oral Administration of nLNP Is Safe for Mice

**Figure 7:**
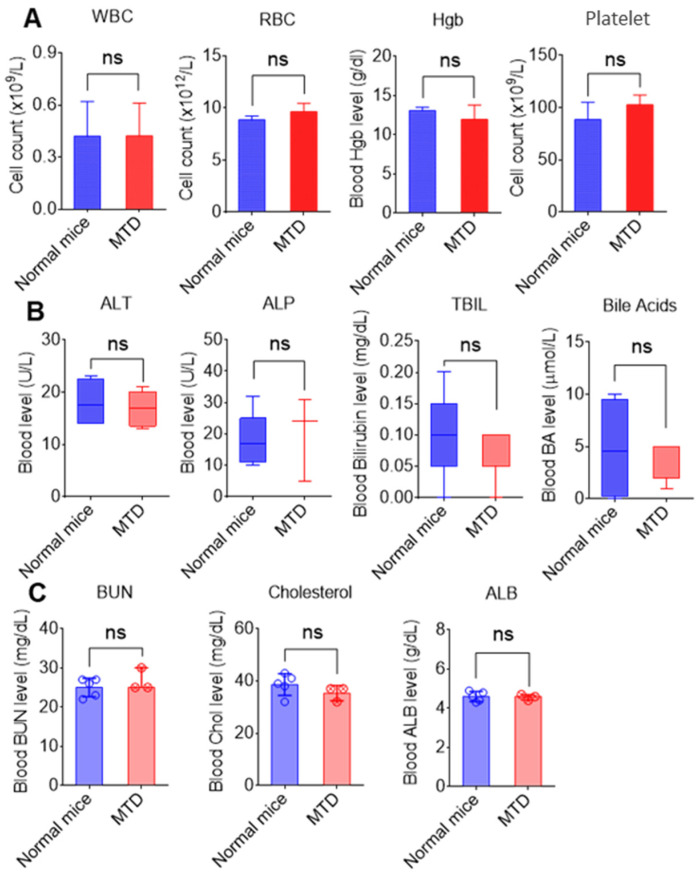
Hematological and biochemical analysis of CD-1 mice from Control and nLNP groups (n = 4). Blood was collected from the retro-orbital sinus, and 25–50 μL of whole blood was used for hematological analyses (VetScan HM5; Abaxis, CA, USA), and 100 µL was used for biochemical analysis (VetScan VS2; Abaxis, CA, USA). The following hematologic parameters are shown: (**A**) Blood counts and hemoglobin: WBC-white blood cells; RBC-red blood cells; HGB-hemoglobin; (**B**) Liver enzymes: ALT-alanine aminotransferase; ALP-alkaline phosphatase; TBIL-total bilirubin; (**C**) Biochemical parameters: BUN-urea nitrogen; ALB-albumin. All results are shown as means ± SD (n = 4, ns: non-significant).

To complement our in vitro findings and ensure a more thorough safety evaluation, we conducted a maximum tolerated dose (MTD) study in CD-1 mice. Ten-week-old female mice were orally administered with nLNP suspension (200 mg/kg) or 1xPBS (control) daily for five consecutive days, with a total accumulated dosage of 1000 mg/kg nLNP, followed by a seven-day monitoring period with regular water and diet. All nLNP-treated mice survived the treatment period, and no differences in activity were observed between the nLNP and control groups during the seven-day post-dose observation. Additionally, the two groups had no significant differences in body weight changes. After sacrificing the mice on day 12, hematological and biochemical indexes were measured. The results demonstrated no significant differences in blood cell counts (Figure 7A), hemoglobin levels, or enzymes reflecting hepatic (Figure 7B) and renal function (BUN, Figure 7C), as well as lipid profile and blood albumin levels (Figure 7C), between nLNP-treated mice and controls. These findings indicate that nLNP is a safe oral drug delivery platform for further development and potential therapeutic applications. 

## 4. Discussion

Oral delivery is a preferred strategy for targeting inflamed intestinal regions in IBD, offering the advantage of more localized drug action compared to other methods like intravenous or rectal administration [32]. This approach is not only less invasive but also more convenient. Plant-derived exosomal lipid nanoparticles have emerged as a promising option for delivering nucleic acids orally in IBD treatment [32,33]. However, these LNPs also contain other components—such as proteins, peptides, nucleic acids, and plant metabolites—that have yet to be fully identified and characterized, posing challenges for large-scale production and quality control under good manufacturing practices (GMP). To address these limitations, high-purity plant-derived lipids can be reassembled into LNPs that replicate the nanostructure of plant exosomal lipid nanoparticles. We reviewed the lipid profiles of literature-reported plant-derived exosomal nanoparticles. Many of these, including grapefruit [34], sesames leaf [35], tea leaf [36], tea flower [37], and turmeric [38], contain very complex lipid profiles, while ginger has a much simpler lipid profile, consisting just three major lipids (DGDG, MGDG, and PA), which together make up more than 90% of the total lipid content. We then used these three lipids to formulate an LNP that mimics the properties of GDNPs. This refined approach retains the critical benefits of ginger-derived nanoparticles, such as precise colon tissue targeting and stability in the gastrointestinal tract, while offering a more practical solution for therapeutic development inspired by nature.

Recent studies have focused on characterizing the tissue-specific targeting capabilities of plant-derived exosomal lipid nanoparticles from carrots, ginger, grapefruit, lemons, tomatoes, and other edible plants. Many of these studies focused on the general uptake and localization of nanoparticles in tissues, providing valuable insights. However, these studies were typically conducted in diseased or healthy animal models without comprehensively comparing nanoparticle uptake between healthy and diseased states. In contrast, our study advanced this research by performing cell-specific targeting analyses and comparing different cell uptake behaviors between inflamed and healthy mice. This offers a more precise and accurate understanding of how nLNP interacts with various cell types in the inflamed intestinal environment. We demonstrated that inflammation significantly enhances the uptake of nLNP by macrophages that have migrated into the mucosa. This increased uptake is likely due to the immune response, as inflammation recruits macrophages to sites of injury, where they are more actively engaged in phagocytosis and immune regulation. Since macrophages play a crucial role in clearing debris and pathogens during inflammation, they exhibit heightened internalization of nLNP in inflamed tissues [39,40]. The significant uptake of nLNP by inflamed colonic macrophages is crucial for the delivery of anti-inflammatory agents, which could reduce the secretion of pro-inflammatory cytokines from macrophages involved in developing ulcerative colitis.

In addition to efficacy, safety evaluation is a critical step in drug development, designed to predict potential adverse effects and mitigate clinical liabilities early in the development process [41]. Safety assessments are essential for the active pharmaceutical ingredient (API) and crucial for the drug delivery system itself, a factor often overlooked. While API safety has traditionally been the primary focus, the evolving recognition of the importance of drug delivery system safety is increasingly acknowledged, especially with the advancement of novel delivery technologies. In this context, we conducted a comprehensive safety evaluation of nLNP, including the InVEST panel, PBMC-based immunosafety assays, and a maximum tolerated dose challenge. Moreover, our previous study revealed that oral administration of nLNP had no noticeable impact on gut microbiota composition 25, supporting the safety profile of this delivery system.

While our study provides valuable insights into the cell-specific targeting capabilities of nLNP in both inflamed and healthy mice, several limitations should be acknowledged. For example, our research primarily focused on an acute colitis model, which may need to fully capture the complexities of the human gastrointestinal environment in ulcerative colitis. Future studies should include chronic inflammation models and consider more diverse in vivo models to enhance the translational potential of these findings. 

## 5. Conclusions

We introduced a novel and targeted approach for treating UC using an orally delivered nLNP. Formulated with three key lipids derived from ginger-exosomal nanoparticles (DGDG, MGDG, and PA), this nLNP retains the nanostructure of ginger-exosomal nanoparticles, providing a safe and effective drug delivery system that specifically targets inflamed colonic tissue. Our findings demonstrate that nLNP is internalized by epithelial cells and lamina propria macrophages in healthy mice. In inflamed mice, the significantly higher migration of macrophages to the epithelium resulted in enhanced nLNP uptake by colonic macrophages compared to healthy mice. This positions nLNP as a promising macrophage-targeted treatment for inflammatory conditions. Importantly, nLNP exhibited excellent safety profiles, with no adverse immunotoxic effects observed in both in vitro and in vivo studies. Altogether, our nLNP represents a safe and efficient solution for delivering therapeutic agents directly to colonic cells, advancing treatment strategies for UC, and offering new avenues for therapeutic development in IBD.

## Figures and Tables

**Table 1:** nLNP’s inhibition assay on InVEST panel.

Target	% Activity Data 1; Data 2		IC (50)	Reference Compound
A1 adenosine	99	103	2.61 × 10^−9^	CPDPX
A2A adenosine	100	101	5.69 × 10^−8^	CGS 21680
alpha1A adrenergic	108	108	9.27 × 10^−9^	prazocin
alpha2A adrenergic	115	116	1.15 × 10^−8^	RX 821002
beta 1 adrenergic	103	103	1.02 × 10^−8^	alprenolol
CCK	90	92	3.78 × 10^−9^	lorglumide
D1 dopamine	106	101	9.01 × 10^−10^	SCH 23390
D3 dopamine	98	100	1.36 × 10^−9^	7-OH DPAT
Muscarinic M1	100	99	4.94 × 10^−9^	pirenzepine
Muscarinic M2	122	110	1.79 × 10^−8^	AF-DX 384
Muscarinic M3	100	97	1.11 × 10^−8^	4-DAMP
5-HT1A	99	98	3.71 × 10^−9^	8-OH DPAT
5-HT1B	100	99	4.85 × 10^−9^	GR125743
5-HT2A	99	96	4.66 × 10^−9^	ketanserin
5-HT3	94	98	1.09 × 10^−9^	GR 65630
Central BZD	103	101	2.24 × 10^−9^	flumazenil
GABA-A	103	107	3.00 × 10^−9^	bicuculline
NMDA	96	98	7.97 × 10^−9^	MK-801
Thrombin alpha	104	105	2.26 × 10^−6^	gabexate mesylate
PDE3A	80	74	2.21 × 10^−5^	IBMX
PDE4A	101	101	3.00 × 10^−5^	IBMX
CYP1A2	100	100	5.82 × 10^−7^	furafylline
CYP2C9	98	96	3.41 × 10^−6^	ketoconazole
CYP2D6	94	104	5.19 × 10^−6^	ketoconazole
CYP3A4	134	126	5.19 × 10^−6^	ketoconazole
CYP19A	96	98	1.70 × 10^−9^	letrozole
AhR-activation	91	96	1.05 × 10^−8^	FICZ
AhR-inhibition	101	101	2.90 × 10^−7^	GNF-351 + 10 nM FICZ

## Data Availability

The data underlying this article will be shared at reasonable request to the corresponding author.

## References

[1] Guan Q. (2019). A Comprehensive Review and Update on the Pathogenesis of Inflammatory Bowel Disease. J. Immunol. Res..

[2] Fakhoury M., Negrulj R., Mooranian A., Al-Salami H. (2014). Inflammatory bowel disease: Clinical aspects and treatments. J. Inflamm. Res..

[3] Wang R., Li Z., Liu S., Zhang D. (2023). Global, regional and national burden of inflammatory bowel disease in 204 countries and territories from 1990 to 2019: A systematic analysis based on the Global Burden of Disease Study 2019. BMJ Open.

[4] Kaplan G.G. (2015). The global burden of IBD: From 2015 to 2025. Nat. Rev. Gastroenterol. Hepatol..

[5] Turpin W., Goethel A., Bedrani L., Croitoru K. (2018). Determinants of IBD Heritability: Genes, Bugs, and More. Inflamm. Bowel Dis..

[6] Aslam N., Lo S.W., Sikafi R., Barnes T., Segal J., Smith P.J., Limdi J.K. (2022). A review of the therapeutic management of ulcerative colitis. Therap. Adv. Gastroenterol..

[7] Bai J., Wang Y., Li F., Wu Y., Chen J., Li M., Wang X., Lv B. (2024). Research advancements and perspectives of inflammatory bowel disease: A comprehensive review. Sci. Prog..

[8] Muzammil M.A., Fariha F., Patel T., Sohail R., Kumar M., Khan E., Khanam B., Kumar S., Khatri M., Varrassi G. (2023). Advancements in Inflammatory Bowel Disease: A Narrative Review of Diagnostics, Management, Epidemiology, Prevalence, Patient Outcomes, Quality of Life, and Clinical Presentation. Cureus.

[9] Perrotta C., Pellegrino P., Moroni E., De Palma C., Cervia D., Danelli P., Clementi E. (2015). Five-aminosalicylic Acid: An update for the reappraisal of an old drug. Gastroenterol. Res. Pract..

[10] Muller A.F., Stevens P.E., McIntyre A.S., Ellison H., Logan R.F. (2005). Experience of 5-aminosalicylate nephrotoxicity in the United Kingdom. Aliment. Pharmacol. Ther..

[11] Singh A., Mahajan R., Kedia S., Dutta A.K., Anand A., Bernstein C.N., Desai D., Pai C.G., Makharia G., Tevethia H.V. (2022). Use of thiopurines in inflammatory bowel disease: An update. Intest. Res..

[12] Gisbert J.P., González-Lama Y., Maté J. (2007). Thiopurine-induced liver injury in patients with inflammatory bowel disease: A systematic review. Am. J. Gastroenterol..

[13] Hadam J., Aoun E., Clarke K., Wasko M.C. (2014). Managing risks of TNF inhibitors: An update for the internist. Clevel. Clin. J. Med..

[14] Xie X., Li F., Chen J.-W., Wang J. (2014). Risk of tuberculosis infection in anti-TNF-α biological therapy: From bench to bedside. J. Microbiol. Immunol. Infect..

[15] Calip G.S., Lee W.-J., Lee T.A., Schumock G.T., Chiu B.C.-H. (2015). Risk of Non-Hodgkin Lymphoma Following Treatment of Inflammatory Conditions with Tumor Necrosis Factor-Alpha Inhibitors. Blood.

[16] Yang C., Zhang M., Lama S., Wang L., Merlin D. (2020). Natural-lipid nanoparticle-based therapeutic approach to deliver 6-shogaol and its metabolites M2 and M13 to the colon to treat ulcerative colitis. J. Control. Release.

[17] Zhang Y., Wang L., Wang Z.-D., Zhou Q., Zhou X., Zhou T., Guan Y.-X., Liu X. (2023). Surface-anchored microbial enzyme-responsive solid lipid nanoparticles enabling colonic budesonide release for ulcerative colitis treatment. J. Nanobiotechnol..

[18] Mitchell M.J., Billingsley M.M., Haley R.M., Wechsler M.E., Peppas N.A., Langer R. (2021). Engineering precision nanoparticles for drug delivery. Nat. Rev. Drug Discov..

[19] Ashfaq R., Rasul A., Asghar S., Kovács A., Berkó S., Budai-Szűcs M. (2023). Lipid Nanoparticles: An Effective Tool to Improve the Bioavailability of Nutraceuticals. Int. J. Mol. Sci..

[20] Yang C., Merlin D. (2020). Can naturally occurring nanoparticle-based targeted drug delivery effectively treat inflammatory bowel disease?. Expert. Opin. Drug Deliv..

[21] Yu L., Deng Z., Liu L., Zhang W., Wang C. (2020). Plant-Derived Nanovesicles: A Novel Form of Nanomedicine. Front. Bioeng. Biotechnol..

[22] Ye L., Wang Y., Xiao F., Wang X., Li X., Cao R., Zhang J., Zhang T.F. (2023). Prausnitzii-derived extracellular vesicles attenuate experimental colitis by regulating intestinal homeostasis in mice. Microb. Cell Fact..

[23] Balboni A., Ailuno G., Baldassari S., Drava G., Petretto A., Grinovero N., Cavalleri O., Angeli E., Lagomarsino A., Canepa P. (2024). Human glioblastoma-derived cell membrane nanovesicles: A novel, cell-specific strategy for boron neutron capture therapy of brain tumors. Sci. Rep..

[24] Mougenot M.F., Pereira V.S., Costa A.L.R., Lancellotti M., Porcionatto M.A., da Silveira J.C., de la Torre L.G. (2022). Biomimetic Nanovesicles—Sources, Design, Production Methods, and Applications. Pharmaceutics.

[25] Sung J., Alghoul Z., Long D., Yang C., Merlin D. (2022). Oral delivery of IL-22 mRNA-loaded lipid nanoparticles targeting the injured intestinal mucosa: A novel therapeutic solution to treat ulcerative colitis. Biomaterials.

[26] Long D., Yang C., Sung J., Merlin D. (2021). Atomic Force Microscopy to Characterize Ginger Lipid-Derived Nanoparticles (GLDNP). Bio-Protocol.

[27] Mow R.J., Srinivasan A., Bolay E., Merlin D., Yang C. (2024). Fluorescent Labeling and Imaging of IL-22 mRNA-Loaded Lipid Nanoparticles. Bio-Protocol.

[28] Morral C., Ghinnagow R., Karakasheva T., Zhou Y., Thadi A., Li N., Yoshor B., Soto G., Chen C., Aleynick D. (2023). Isolation of Epithelial and Stromal Cells from Colon Tissues in Homeostasis and Under Inflammatory Conditions. Bio-Protocol.

[29] Kuczma M.P., Szurek E.A., Cebula A., Chassaing B., Jung Y.-J., Kang S.-M., Fox J.G., Stecher B., Ignatowicz L. (2020). Commensal epitopes drive differentiation of colonic T(regs). Sci. Adv..

[30] Zhang M., Viennois E., Prasad M., Zhang Y., Wang L., Zhang Z., Han M.K., Xiao B., Xu C., Srinivasan S. (2016). Edible ginger-derived nanoparticles: A novel therapeutic approach for the prevention and treatment of inflammatory bowel disease and colitis-associated cancer. Biomaterials.

[31] Wardill H.R., Choo J.M., Dmochowska N., Mavrangelos C., Campaniello M.A., Bowen J.M., Rogers G.B., Hughes P.A. (2019). Acute Colitis Drives Tolerance by Persistently Altering the Epithelial Barrier and Innate and Adaptive Immunity. Inflamm. Bowel Dis..

[32] Yang C., Sharma K., Mow R.J., Bolay E., Srinivasan A., Merlin D. (2024). Unleashing the Potential of Oral Deliverable Nanomedicine in the Treatment of Inflammatory Bowel Disease. Cell. Mol. Gastroenterol. Hepatol..

[33] Yang C., Alpini G., Glaser S., Merlin D. (2022). Lipid nanoparticles for oral delivery of nucleic acids for treating inflammatory bowel disease. Nanomedicine.

[34] Wang Q., Zhuang X., Mu J., Deng Z.B., Jiang H., Zhang L., Xiang X., Wang B., Yan J., Miller D. (2013). Delivery of Therapeutic Agents by Nanoparticles Made of Grapefruit-Derived Lipids. Nat. Commun..

[35] Jiang D., Li Z., Liu H., Liu H., Xia X., Xiang X. (2024). Plant exosome-like nanovesicles derived from sesame leaves as carriers for luteolin delivery: Molecular docking, stability, and bioactivity. Food Chem..

[36] Zu M., Xie D., Canup B.S., Chen N., Wang Y., Sun R., Zhang Z., Fu Y., Dai F., Xiao B. (2021). ‘Green’ nanotherapeutics from tea leaves for orally targeted prevention and alleviation of colon diseases. Biomaterials.

[37] Chen Q., Li Q., Liang Y., Zu M., Chen N., Canup B.S., Luo L., Wang C., Zeng L., Xiao B. (2022). Natural exosome-like nanovesicles from edible tea flowers suppress metastatic breast cancer via ROS generation and microbiota modulation. Acta Pharm. Sin. B.

[38] Gao C., Zhou Y., Chen Z., Li H., Xiao Y., Hao W., Zhu Y., Vong C.T., Farag M.A., Wang Y. (2022). Turmeric-derived nanovesicles as novel nanobiologics for targeted therapy of ulcerative colitis. Theranostics.

[39] Hu G., Guo M., Xu J., Wu F., Fan J., Huang Q., Yang G., Lv Z., Wang X., Jin Y. (2019). Nanoparticles Targeting Macrophages as Potential Clinical Therapeutic Agents Against Cancer and Inflammation. Front. Immunol..

[40] You D.J., Lee H.Y., Bonner J.C., Bonner J.C., Brown J.M. (2020). Macrophages: First Innate Immune Responders to Nanomaterials. Interaction of Nanomaterials with the Immune System.

[41] Bendels S., Bissantz C., Fasching B., Gerebtzoff G., Guba W., Kansy M., Migeon J., Mohr S., Peters J.-U., Tillier F. (2019). Safety screening in early drug discovery: An optimized assay panel. J. Pharmacol. Toxicol. Methods.

